# Case Report: cytoreductive prostatectomy after platinum–etoposide chemotherapy for *de novo* metastatic prostatic small cell neuroendocrine carcinoma

**DOI:** 10.3389/fruro.2026.1934905

**Published:** 2026-09-16

**Authors:** Yanxin Wang, Qiwu Wang, Hang Yang

**Affiliations:** Chinese People’s Liberation Army Western Theater General Hospital, Chengdu, China

**Keywords:** case report, cytoreductive surgery, etoposide plus cisplatin, neuroendocrine prostate cancer, prostatic small cell neuroendocrine carcinoma

## Abstract

**Objective:**

To describe the diagnostic challenges, treatment response, and potential benefits and risks of cytoreductive surgery in *de novo* metastatic prostatic small cell neuroendocrine carcinoma (SCNEC).

**Methods:**

We retrospectively reviewed the clinical course of one patient with *de novo* metastatic prostatic SCNEC.

**Results:**

A 50-year-old man presented in June 2022 with progressive dysuria for more than 1 year and acute urinary retention for 1 day. Transurethral resection of the prostate (TURP) was performed to relieve obstruction, and review of the specimen supported SCNEC. Staging ^18^F-FDG PET/CT showed an FDG-avid prostate, multiple lymph nodes, and bilateral non-FDG-avid pulmonary nodules without a dominant pulmonary primary; a prostatic primary with clinically suspected pulmonary metastases was favored, although the lung lesions were not biopsied. Seven cycles of etoposide plus cisplatin (EP) produced marked local regression, including a reduction in prostate volume from 72 to 17 cm³, but residual tumor persisted and a RECIST response category could not be assigned retrospectively. Cytoreductive laparoscopic radical prostatectomy was performed in February 2023. Postoperative pathology showed a mixed neuroendocrine–non-neuroendocrine neoplasm (MiNEN). A vesicourethral anastomotic leak improved conservatively, and urinary obstruction improved for approximately 3 months. Disease progressed in July 2023, and irinotecan plus carboplatin provided no durable clinical disease control. The patient died in December 2023, approximately 18 months after diagnosis.

**Conclusion:**

EP can produce substantial but temporary regression in metastatic prostatic SCNEC. This case did not establish a survival benefit from surgery, although temporary relief of urinary obstruction was reported. Systemic therapy should remain the priority, and surgery should be limited to carefully selected local-control or palliative indications.

## 1 Introduction

Prostate cancer accounted for 7.3% of all newly diagnosed cancers worldwide in 2022 and ranked fourth among cancers overall (1). Conventional acinar adenocarcinoma accounts for the great majority of prostatic malignancies, whereas *de novo* primary neuroendocrine prostate cancer is rare: a SEER analysis identified 309 cases among 510,913 prostate cancers (0.06%), of which 186 (60.2%) had small-cell histology (2). The 2022 WHO classification includes well-differentiated neuroendocrine tumor, small cell and large cell neuroendocrine carcinomas, and mixed neuroendocrine neoplasms in its genitourinary neuroendocrine taxonomy, and recognizes treatment-related neuroendocrine prostatic carcinoma as a distinct prostatic tumor type (3). Outcomes are poor: a retrospective case series of patients with metastatic prostatic small-cell carcinoma treated with chemotherapy reported a median survival of 9.4 months after diagnosis (4), while a population-based cohort reported observed survival rates of 47.9% at 12 months and 14.3% at 60 months (5).

Treatment-emergent small cell neuroendocrine prostate cancer (t-SCNEC) is a recognized phenotype of metastatic castration-resistant prostate cancer after androgen-receptor-directed treatment; a prospective metastatic-biopsy study detected t-SCNEC in 17% of evaluable patients (6). In contrast, *de novo* SCNEC is present at the initial cancer diagnosis, before exposure to androgen deprivation therapy (ADT) or androgen-receptor pathway inhibitors. *De novo* SCNEC typically presents with normal or mildly elevated PSA levels and is often AR-independent, whereas treatment-emergent disease develops under the selective pressure of prior androgen-receptor-directed therapy (7).

Because prospective trials are lacking, the treatment paradigm for prostatic SCNEC is largely extrapolated from small-cell lung cancer because of their similar histologies. Platinum-based chemotherapy combined with etoposide is commonly used in this setting (7). In a retrospective case series of 21 patients with metastatic prostatic small-cell carcinoma treated with chemotherapy regimens that included etoposide plus cisplatin, 13 patients (62%) responded and median survival after diagnosis was 9.4 months; these results cannot be interpreted as EP-specific efficacy because several regimens were used (4). The role of cytoreductive surgery in patients presenting with distant metastases remains undefined. The present case is clinically relevant because it illustrates diagnostic uncertainty created by TTF-1 positivity and unbiopsied pulmonary nodules, a marked but incomplete response to EP, temporary symptom relief after cytoreductive prostatectomy, and rapid subsequent progression. It therefore informs patient selection and counseling without implying that surgery prolonged survival.

## 2 Case report

### 2.1 Patient information

Written informed consent was obtained from the patient for the publication of this case report and any accompanying images. A copy of the written consent is available for review by the Editor-in-Chief of this journal.

Presentation and initial management: On June 17, 2022, a 50-year-old man was admitted to Chinese People’s Liberation Army Western Theater General Hospital four days after undergoing TURP at a local hospital. He had initially presented with progressive dysuria for more than 1 year and acute urinary retention for 1 day. The available local-hospital evaluation showed a serum PSA level of 2.06 ng/mL, prostatic enlargement on digital rectal examination, a prostate volume of 93 cm³ on ultrasonography, and an enlarged prostate with heterogeneous signal intensity and multiple enlarged lymph nodes along the bilateral iliac vessels and within the pelvis on pelvic MRI; contrast-enhanced MRI was recommended. The outpatient records did not document the exact dates of these local-hospital investigations; the available record places them within the interval between two documented outpatient follow-up visits at the same institution. TURP was performed as a deobstructive procedure to relieve urinary retention and persistent voiding difficulty, rather than as definitive surgery. Histopathologic examination after TURP initially suggested prostatic adenocarcinoma, prompting referral for further evaluation.

Relevant history and symptoms: The patient had no smoking history, no known or suspected pulmonary disease, and no pulmonary symptoms. He reported no bone pain. There was no family history of prostate cancer and no history of androgen deprivation therapy (ADT).

Physical examination on admission: Vital signs were stable. Digital rectal examination revealed an enlarged prostate with a hard texture, nodular surface, and no tenderness.

Laboratory tests: On June 17, 2022, the serum PSA level at the same institution was 2.76 ng/mL (reference value <4.0 ng/mL), distinct from the pre-TURP value of 2.06 ng/mL measured at the local hospital. The testosterone level was within the reference range, and routine blood tests and liver and kidney function tests were unremarkable.

### 2.2 Imaging examinations

The available pre-TURP pelvic MRI showed an enlarged heterogeneous prostate and multiple enlarged iliac and pelvic lymph nodes. On June 27, 2022, bone scintigraphy showed nonspecific mild uptake in the right shoulder, left wrist, and left knee. After TURP supported SCNEC, ^18^F-FDG PET/CT on July 1, 2022, showed heterogeneous prostatic uptake after TURP, possible seminal-vesicle involvement, FDG-avid pelvic/systemic lymph nodes, and scattered bilateral pulmonary nodules without abnormal uptake or a dominant pulmonary mass (**Figure 1**). Possible distal right-ureter involvement with hydronephrosis was also reported. The record documented T4N1M1c, although T4 reflected suspected adjacent-organ involvement and M1c clinically suspected, unbiopsied pulmonary metastases; neither was histologically confirmed.

**Figure 1:**
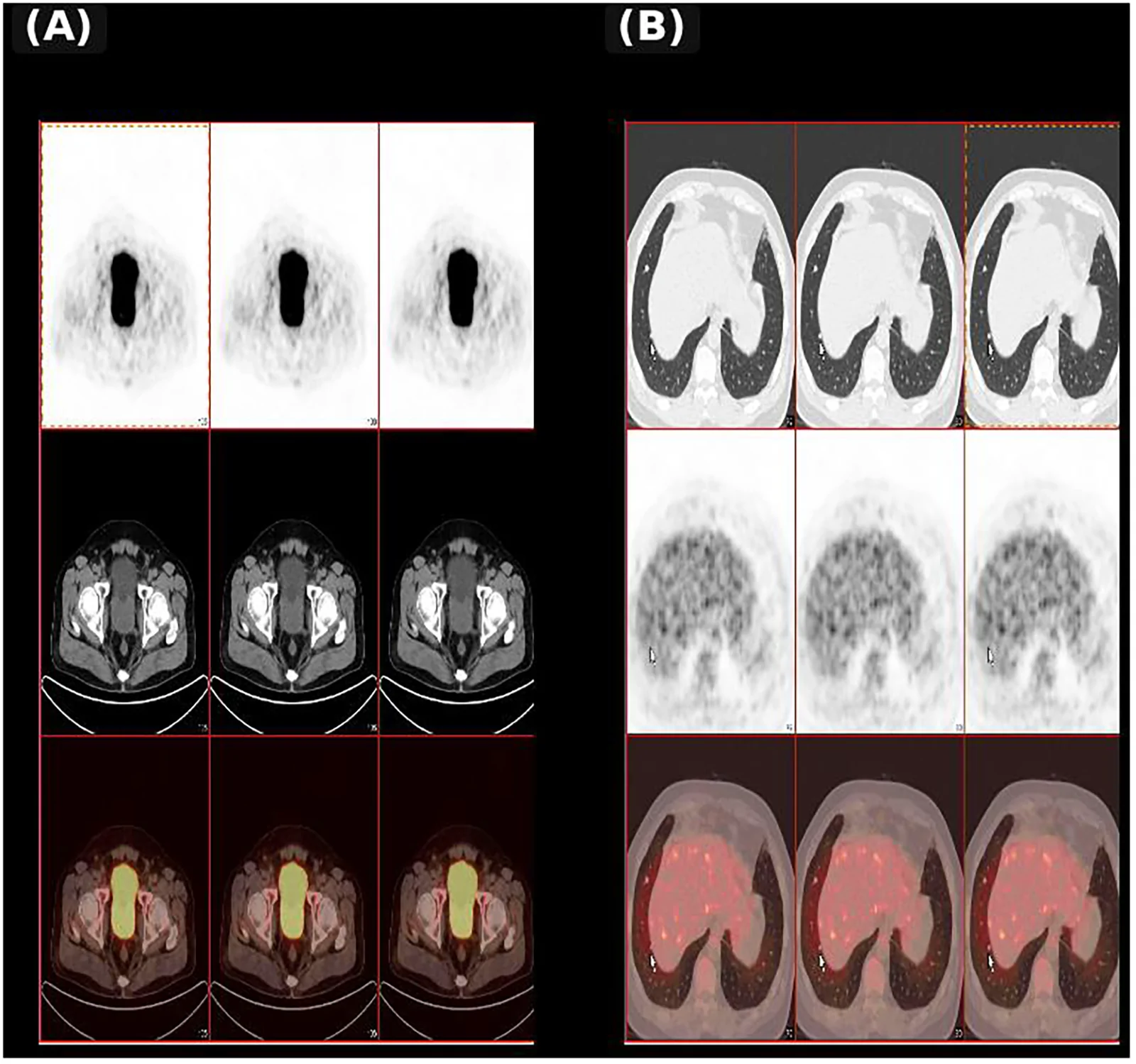
^18^F-FDG PET/CT images from the reported patient obtained on July 1, 2022, after the TURP specimen supported SCNEC. **(A)** Enlarged prostate with heterogeneous increased FDG uptake after TURP. **(B)** Multiple bilateral pulmonary nodules on CT without abnormal FDG uptake or a dominant pulmonary primary lesion. The pulmonary nodules were clinically considered possible metastases but were not histologically confirmed. PET/CT was used for staging and did not establish the histopathologic diagnosis.

### 2.3 Pathological examination

On June 27, 2022, review of the TURP specimen from the local hospital showed morphologic and immunohistochemical features supporting SCNEC. The immunophenotype was AR(−), CD56(+), CDX2(−), CK(+), CK20(−), CK5/6(−), CK7 (focally +), CgA(+), Ki-67 (approximately 50%), P504S(+), p63(−), PSA(−), Syn(+), and TTF-1(+) (**Figure 2**).

**Figure 2:**
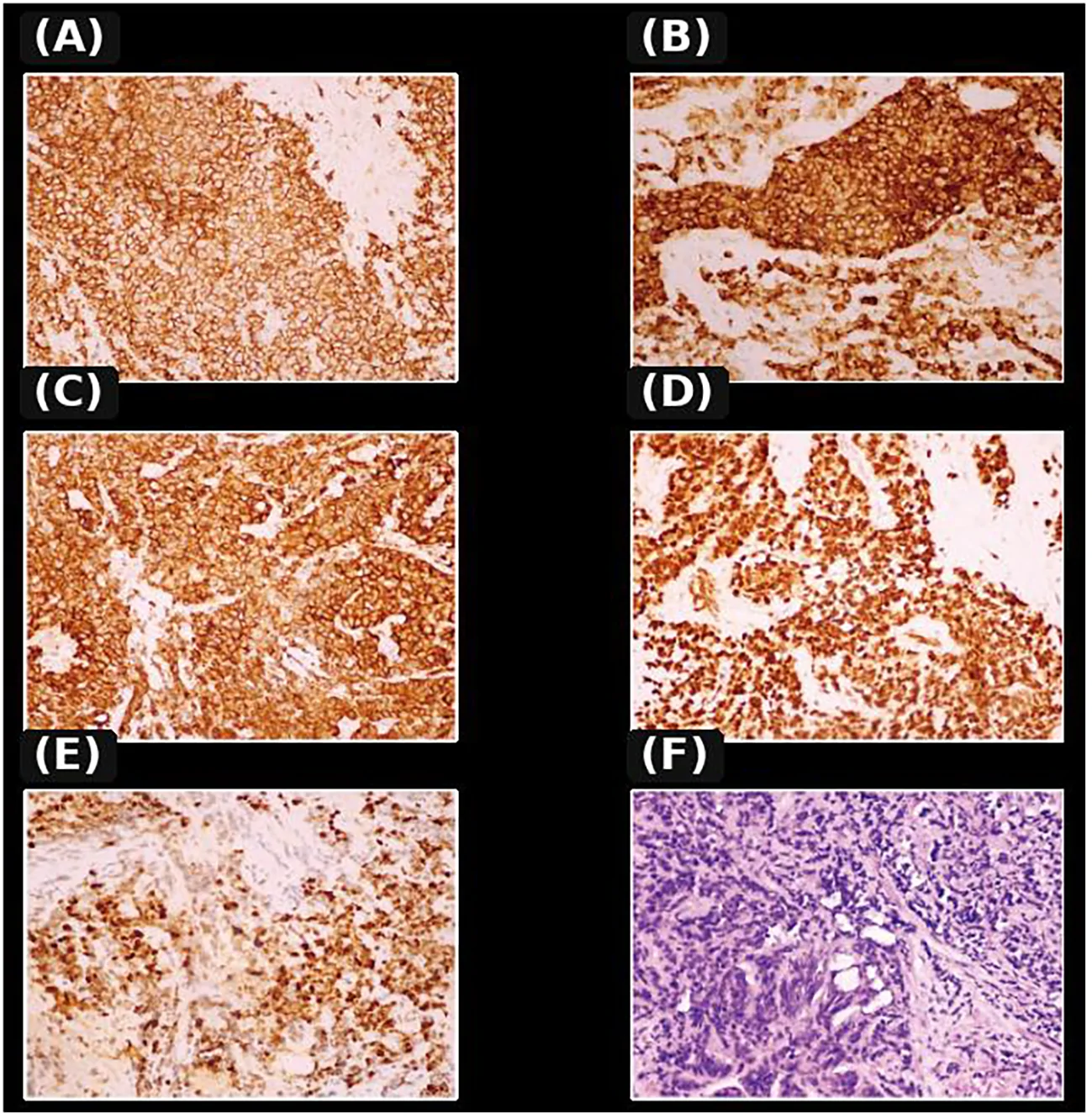
Representative histopathologic and immunohistochemical findings in the TURP specimen. **(A)** Diffuse membranous CD56 expression (original magnification, ×40). **(B)** Chromogranin A expression (×40). **(C)** Diffuse synaptophysin expression (×40). **(D)** Nuclear TTF-1 expression (×40). **(E)** Ki-67 labeling in approximately 50% of tumor cells (×40). **(F)** Hematoxylin and eosin staining showing a poorly differentiated small-cell neoplasm (×40). The combined morphology and neuroendocrine-marker profile supported SCNEC; TTF-1 positivity alone did not determine the site of origin.

The diagnosis of SCNEC in the TURP specimen was based on the small-cell morphology together with expression of the neuroendocrine markers Syn, CgA, and CD56 and a Ki-67 proliferation index of approximately 50%; TTF-1 positivity was not used as a stand-alone diagnostic criterion. TTF-1 may be expressed in extrapulmonary small-cell carcinomas and was positive in 23 of 44 (52.3%) prostatic small-cell carcinomas in a large pathologic series; therefore, TTF-1 positivity neither proves a pulmonary primary nor determines the site of origin (8, 9). In this case, pulmonary small-cell carcinoma metastatic to the prostate remained a differential diagnosis because bilateral pulmonary nodules were present. A prostatic primary was favored because the presenting symptoms and dominant mass effect arose from the prostate, the patient had no smoking history or pulmonary symptoms, and PET/CT showed multiple scattered non-FDG-avid pulmonary nodules without a dominant pulmonary mass. A pulmonary nodule biopsy was recommended but declined. Accordingly, the integrated clinicopathologic diagnosis was *de novo* primary prostatic SCNEC with clinically suspected pulmonary metastases; however, a pulmonary primary could not be excluded with absolute certainty in the absence of histologic sampling of the lung lesions.

Histopathologic examination of the radical prostatectomy specimen on February 23, 2023, demonstrated both small cell neuroendocrine carcinoma and poorly differentiated non-neuroendocrine (adenocarcinoma) components and was reported as a mixed neuroendocrine–non-neuroendocrine neoplasm (MiNEN). Vascular and perineural invasion were present, and the Ki-67 proliferation index was 60%–70%. Neither seminal vesicle involvement nor a positive surgical margin was identified. The difference between the TURP and prostatectomy findings most likely reflected sampling limitations of TURP and intratumoral heterogeneity. A treatment-related shift in component predominance could not be established from this single case.

### 2.4 Treatment course

#### 2.4.1 First-line chemotherapy (EP regimen)

Because prospective trials are lacking, the treatment paradigm for prostatic SCNEC is largely extrapolated from small-cell lung cancer because of their similar histologies. Platinum-based chemotherapy combined with etoposide is commonly used in this setting (7). EP began June 30, 2022; cycles 2–7 began July 19, August 15, September 16, October 16, November 21, and December 26. Each 28-day cycle comprised etoposide 100 mg/m²/day and cisplatin 25 mg/m²/day intravenously on days 1–3. The chemotherapy course was intermittently complicated by nausea, vomiting, and dysphagia, which resolved with supportive care without the need for dose reduction or treatment delay. Pelvic MRI on July 24 was the first available comparison after EP began; pelvic MRI on August 18 and September 13 showed further reduction in prostatic enhancement and pelvic nodes. After five cycles, pelvic MRI on October 20 showed no definite enhancing focus in most of the prostate, but residual urethral enhancement and small pelvic nodes remained; biopsy on October 24 still showed poorly differentiated carcinoma. After seven cycles, pelvic MRI on December 27 showed persistent right peripheral-zone diffusion restriction and residual disease. February 2023 ultrasonography estimated 17 cm³ versus 72 cm³ at the same institution (approximately 76% reduction); the pre-TURP local-hospital volume was 93 cm³. This documented marked local radiographic response, but absent serial target-lesion measurements and comprehensive restaging, RECIST 1.1 partial response or stable disease could not be assigned (**Figure 3**) (10).

**Figure 3:**
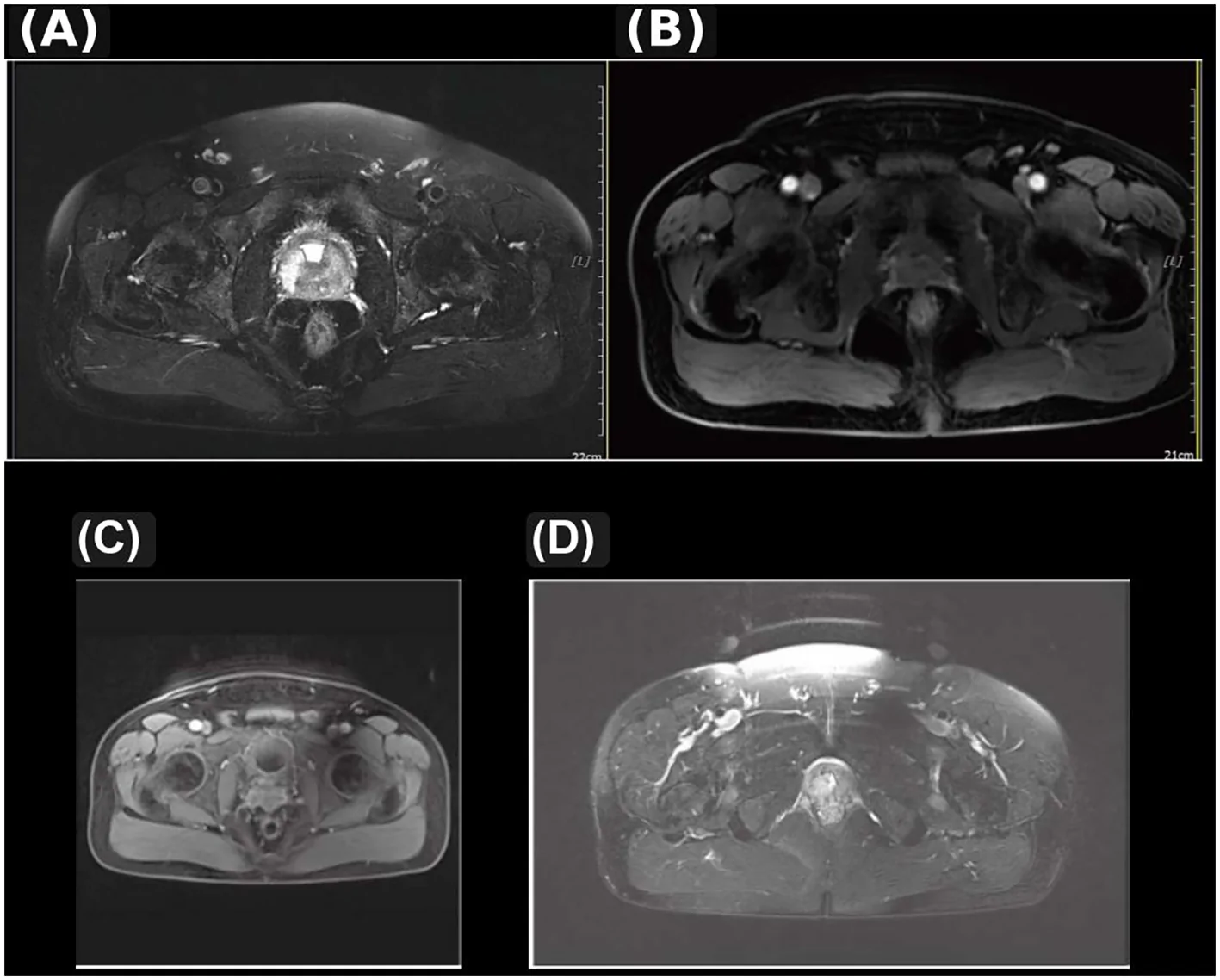
Selected axial pelvic MRI images during first-line EP treatment and after prostatectomy. **(A)** MRI on July 24, 2022, the earliest available comparison study after EP had begun, showing an enlarged heterogeneous prostate. **(B)** MRI on October 20, 2022, after five EP cycles, showing marked reduction in abnormal prostatic enhancement, with residual enhancement around the dilated prostatic urethra. **(C, D)** Postoperative pelvic MRI images obtained in April 2023, approximately 2 months after prostatectomy, showing irregular tissue in the prostatectomy bed with mild adjacent pelvic-floor fascial thickening; the findings were indeterminate for recurrence at that time. The treatment-response images show a marked local radiographic response but do not provide RECIST target-lesion measurements.

#### 2.4.2 Cytoreductive surgery

After seven EP cycles, marked local radiographic response but persistent disease remained. At the patient’s request, a multidisciplinary team considered surgery for local control because of his age, good performance status, persistent tumor burden, obstructive symptoms, and preference for local treatment. Goals were to relieve obstruction, reduce bleeding risk, and decrease local burden; no survival benefit was established. Laparoscopic radical prostatectomy was completed on February 14, 2023, without intraoperative complications. Marked edema and adhesions were encountered. The postoperative course was complicated by a vesicourethral anastomotic leak, which improved after approximately 1 week of drainage and catheterization; no other postoperative complications were documented. Drains were removed and the patient was discharged improved on February 27. The catheter was removed approximately 2 weeks later, with no documented clinically significant incontinence.

#### 2.4.3 Postoperative treatment and progression

The patient was readmitted on April 12, 2023, for postoperative reassessment, including repeat pelvic MRI and other relevant examinations. His first postoperative EP cycle was administered on April 20, 2023, and the second on May 19, 2023. Cystoscopy on April 24 showed no obvious intravesical neoplasm. On July 18, approximately 5 months after surgery, painless gross hematuria, renal failure, bilateral hydronephrosis, and anemia led to clot evacuation and right nephrostomy on July 20; left nephrostomy was initially unsuccessful. Repeat clot evacuation was required July 22 and 24. Non-contrast abdominal CT performed on July 25 showed an irregular patchy hypo-/isodense area in the prostatectomy bed, with an indistinct boundary from the adjacent posterior bladder wall, a difficult-to-measure extent, blurred surrounding fat planes, and mild thickening of the adjacent pelvic-floor fascia. Multiple lymph nodes were identified in the left retroperitoneal and abdominopelvic regions, some of which were mildly enlarged; these findings raised concern for concurrent nodal disease. Bilateral hydroureteronephrosis and possible left-ureter involvement were also reported. The CT findings raised suspicion of local and nodal recurrence, although recurrence was not pathologically confirmed. Left nephrostomy was completed July 27 and repeated August 7 after dislodgement. These events were associated with suspected recurrence and were distinct from the early leak. No objective response category could be assigned to postoperative EP without target measurements.

#### 2.4.4 Second-line chemotherapy and terminal phase

Because of progression after EP, irinotecan plus carboplatin began August 8, 2023 (irinotecan 80 mg/m²/day on days 1 and 8; carboplatin 400 mg/m² on day 2 of each 21-day cycle). Because renal impairment made the patient ineligible for cisplatin, carboplatin was selected for second-line treatment. The patient was admitted on September 13, 2023, because of a urinary tract infection and initially received levofloxacin. Urine culture on September 19 grew Citrobacter freundii, which was resistant to levofloxacin; treatment was therefore changed to amikacin combined with minocycline, with subsequent clinical improvement. The second cycle of irinotecan plus carboplatin was administered on September 26, 2023, followed by single-agent irinotecan on October 10, 2023. No formal restaging was available, so objective response could not be assigned. Clinical deterioration continued, and the family chose best supportive care. The patient died from multiorgan failure in December 2023, approximately 18 months after diagnosis. Progression after platinum exposure cannot distinguish acquired resistance from intrinsic SCNEC aggressiveness; both remain plausible.

### 2.5 Patient perspective

The patient had strongly requested local surgery after chemotherapy because he hoped to obtain better local control and relief of urinary obstruction. During approximately the first 3 months after cytoreductive surgery, he reported that urinary tract obstruction was markedly improved compared with the preoperative period. From the start of chemotherapy through approximately 3 months after surgery, he also reported less pain and better overall quality of life than at initial presentation. These benefits were subjective and not durable, as his condition subsequently worsened with disease progression. Because no validated symptom score or quality-of-life instrument was administered, the magnitude of improvement could not be quantified. The complete clinical course is summarized in **Figure 4**.

**Figure 4:**
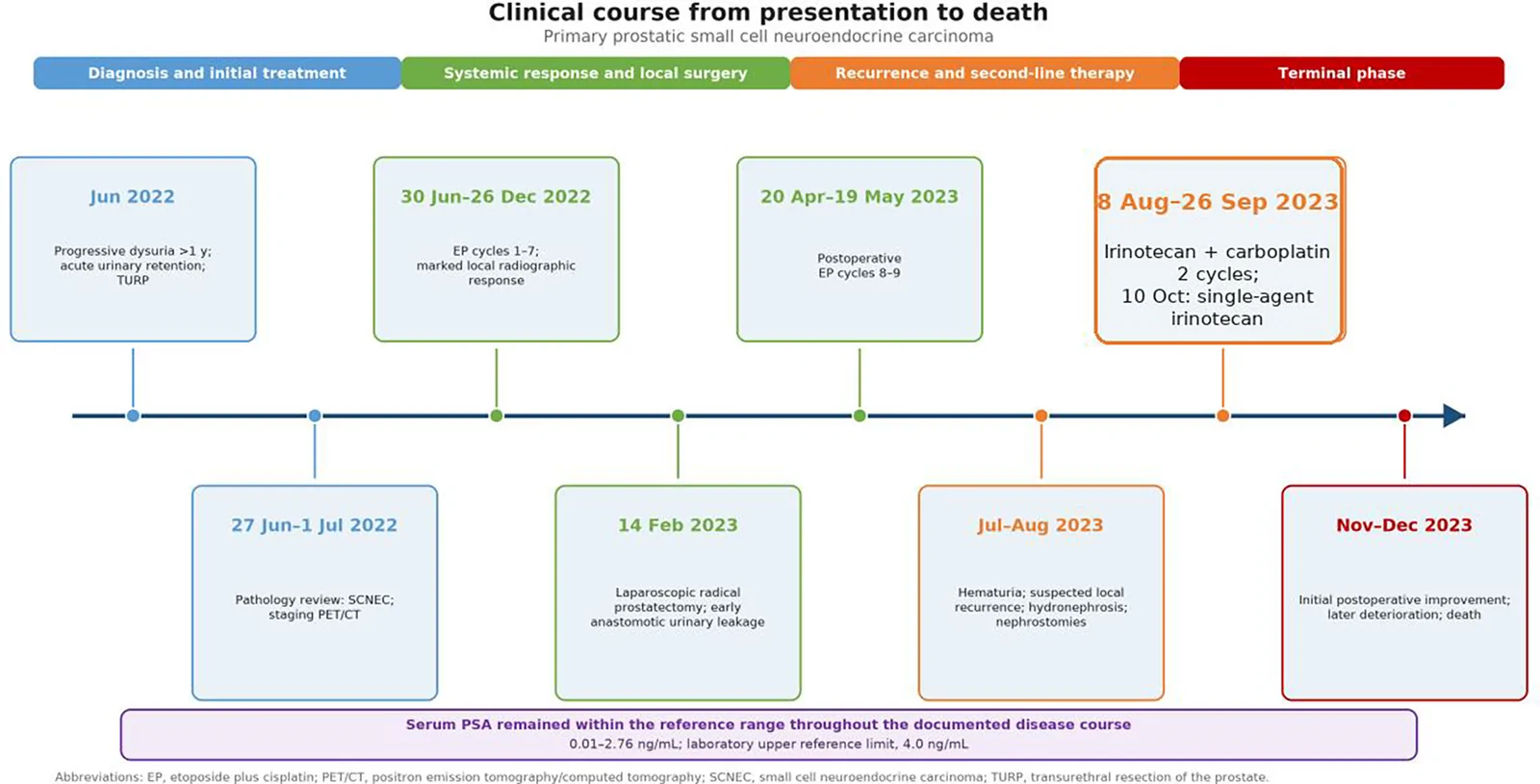
Clinical timeline from initial presentation to death. The timeline summarizes diagnostic assessment, systemic treatment, surgery, early postoperative vesicourethral anastomotic leakage, recurrence-related urinary complications, second-line treatment, and follow-up. The second-line timeline specifies two cycles of irinotecan plus carboplatin (August 8 and September 26, 2023), followed by single-agent irinotecan on October 10, 2023. Serum PSA remained within the laboratory reference range throughout the documented disease course (0.01–2.76 ng/mL; upper reference limit, 4.0 ng/mL). EP, etoposide plus cisplatin; PET/CT, positron emission tomography/computed tomography; SCNEC, small cell neuroendocrine carcinoma; TURP, transurethral resection of the prostate.

## 3 Discussion

SCNEC is a poorly differentiated and rapidly progressive form of neuroendocrine prostate cancer. In the retrospective case series reported by Amato et al., 13 of 21 patients (62%) with metastatic prostatic small-cell carcinoma responded to chemotherapy, but median survival after diagnosis was only 9.4 months (4). In a population-based cohort of 191 patients, 60.5% presented with metastatic disease, and observed survival was 47.9% at 12 months and 14.3% at 60 months (5). Age younger than 60 years, concomitant low-grade adenocarcinoma, and absence of metastasis were favorable prognostic factors in that cohort (5). The present patient survived approximately 18 months from diagnosis. This observation is descriptive and cannot be attributed to surgery; no causal survival inference can be made from a single case without a comparator.

Given the biological similarity between prostatic SCNEC and small-cell lung cancer, current systemic treatment commonly uses platinum-based chemotherapy combined with etoposide. In this patient, seven preoperative EP cycles produced a marked local radiographic response in the prostate and pelvic lymph nodes but did not eradicate viable tumor, as demonstrated by the October 2022 biopsy, the December 2022 pelvic MRI, and the prostatectomy specimen. Progression became clinically apparent after two additional postoperative EP cycles. This temporal pattern may be compatible with acquired platinum resistance; however, the absence of serial systemic target-lesion measurements and the intrinsically aggressive biology of SCNEC prevent resistance from being distinguished from rapid natural disease progression in a single case. We therefore describe the course as progression after EP exposure rather than definitive proof of drug resistance. Irinotecan plus carboplatin and subsequent single-agent irinotecan did not provide durable clinical disease control, but the absence of formal restaging similarly precluded assignment of an objective response category.

Evidence for radical surgery in metastatic SCNEC is extremely limited. In a SEER analysis of 309 *de novo* neuroendocrine prostate cancers, 198 (64.1%) were metastatic; radical prostatectomy was associated with improved survival only in the nonmetastatic subgroup (2). In an NCDB study of 243 nonmetastatic small-cell carcinomas, radiotherapy and surgery had no significant overall-survival difference in pure or mixed histology (11). These findings cannot be extrapolated to extensive M1c disease. Among 379 patients with nodal or distant metastatic small-cell carcinoma, only 3 (1%) underwent radical prostatectomy, precluding a reliable surgical-benefit estimate; chemotherapy, rather than surgery, was associated with longer survival (12). Because these observational cohorts did not provide a reliable metastatic-surgery comparison, and because this single case lacked a counterfactual comparator, the available data cannot quantify any survival benefit from surgery in metastatic patients. Thus, cytoreductive prostatectomy is not an established survival-prolonging treatment. In this case it was undertaken after multidisciplinary review for cytoreduction and symptom control. The early leak improved conservatively, whereas later hematuria, ureteral involvement, hydronephrosis, and nephrostomy reflected suspected recurrence. Urinary obstruction improved for approximately 3 months, but no validated patient-reported instrument was used and benefits were not durable. Systemic therapy should remain the priority; surgery, if considered, requires carefully selected local-control or palliative indications.

The role of androgen deprivation therapy (ADT) depends on tumor phenotype. Pure SCNEC is commonly independent of the androgen receptor (AR) pathway and generally does not meaningfully benefit from ADT; platinum-based chemotherapy remains the backbone (7). ADT may be considered for clinically relevant AR-driven adenocarcinoma in mixed tumors, although supporting data are sparse. In the nonmetastatic NCDB cohort, ADT was not associated with improved survival in pure or mixed histology11. Treatment-emergent SCNEC may arise after AR-directed therapy, and prospective biopsy data document t-SCNEC in mCRPC6. Here SCNEC was present at initial diagnosis without prior ADT or AR-pathway inhibition, supporting *de novo* classification. This does not establish organ of origin; the prostatic primary was favored on integrated clinical, radiologic, and pathologic grounds. PSA- and AR-negative tumor with normal-range PSA did not provide a strong rationale for ADT activity against the dominant SCNEC phenotype. Because AR staining of the non-neuroendocrine component was not separately reported, the potential efficacy of ADT for that component cannot be assessed.

Peptide receptor radionuclide therapy (PRRT) has demonstrated clinical benefit in selected somatostatin-receptor-positive gastroenteropancreatic neuroendocrine tumors; however, these data do not establish efficacy in prostatic SCNEC (13). Future research should focus on the molecular mechanisms of neuroendocrine differentiation, biomarkers for patient selection, and prospective evaluation of systemic treatment strategies rather than assuming benefit from local surgery.

## 4 Conclusion

*De novo* primary prostatic SCNEC is a rare, highly aggressive malignancy that may present with distant metastases and a normal serum PSA level. In this case, EP produced marked but incomplete local tumor regression, cytoreductive prostatectomy was followed by temporary improvement in urinary obstruction, and disease subsequently progressed. The patient survived approximately 18 months from diagnosis. This case does not establish a survival benefit from surgery. Systemic therapy should remain the priority in metastatic SCNEC, and local surgery should be considered only after multidisciplinary review for carefully selected local-control or palliative indications.

## Data Availability

The raw data supporting the conclusions of this article will be made available by the authors, without undue reservation.
