# Supplementary material for: Case Report: cytoreductive prostatectomy after platinum–etoposide chemotherapy for de novo metastatic prostatic small cell neuroendocrine carcinoma
Source: Front Urol. 2026 Sep 16;6:1934905. doi: 10.3389/fruro.2026.1934905 (PMC13623550; doi:10.3389/fruro.2026.1934905)
Supplement: Supplementary file 1 [file Supplementaryfile1.pdf]

2013 CARE Checklist

Completed for Frontiers in Urology Case Report Submission

Case Report: Cytoreductive Prostatectomy After Platinum–Etoposide Chemotherapy for De Novo Metastatic Prostatic Small Cell Neuroendocrine Carcinoma

Page references correspond to the clean second-revision manuscript. Checklist source: CARE Case Report Guidelines (https://www.care-statement.org/checklist).

Item	Topic	CARE checklist requirement	Reported on page/section	Compliance note
**1**	Title	The diagnosis or intervention of primary focus followed by the words 'case report'.	p. 1, title	Reported
**2**	Key words	Two to five key words identifying the diagnosis or intervention, including 'case report'.	p. 1, Keywords	Reported: 5 keywords
**3a**	Abstract	Introduction: what is unique about the case and what it adds to the literature.	pp. 1–2, Objective and Conclusion	Reported
**3b**	Abstract	The patient's main concerns and important clinical findings.	pp. 1–2, Results	Reported
**3c**	Abstract	Primary diagnoses, interventions, and outcomes.	pp. 1–2, Results	Reported
**3d**	Abstract	Conclusion: one or more take-away lessons.	p. 2, Conclusion	Reported
**4**	Introduction	Briefly summarize why the case is unique, with relevant literature.	pp. 2–3, Introduction	Reported
**5a**	Patient information	De-identified patient-specific information.	pp. 3–4, Patient Information	Reported
**5b**	Patient information	Primary concerns and symptoms.	p. 3, Presentation and initial management	Reported
**5c**	Patient information	Medical, family, and psychosocial history, including relevant genetic information.	pp. 3–4, Relevant history and symptoms; p. 7, Patient Perspective	Reported to the extent available; no relevant genetic testing was documented
**5d**	Patient information	Relevant past interventions and their outcomes.	p. 3, local-hospital TURP and referral	Reported

Item	Topic	CARE checklist requirement	Reported on page/section	Compliance note
**6**	Clinical findings	Significant physical examination and important clinical findings.	pp. 3–4, Physical examination and Laboratory tests	Reported
**7**	Timeline	Historical and current information organized as a timeline figure or table.	p. 13, Figure 4	Reported
**8a**	Diagnostic assessment	Diagnostic methods: physical examination, laboratory testing, imaging, surveys.	pp. 3–5, Patient Information, Imaging, and Pathology	Reported
**8b**	Diagnostic assessment	Diagnostic challenges.	pp. 4–5, TTF-1 positivity, pulmonary differential, unbiopsied nodules	Reported
**8c**	Diagnostic assessment	Diagnosis, including other diagnoses considered.	pp. 4–5, Pathological Examination	Reported
**8d**	Diagnostic assessment	Prognostic characteristics, when applicable.	pp. 4–5 and 7, stage, Ki-67, vascular/perineural invasion, prognostic context	Reported
**9a**	Therapeutic intervention	Types of intervention: pharmacologic, surgical, preventive.	pp. 5–7, Treatment Course	Reported
**9b**	Therapeutic intervention	Administration: dosage, strength, and duration.	pp. 5–7, EP and irinotecan/carboplatin schedules	Reported
**9c**	Therapeutic intervention	Changes in interventions, with explanations.	pp. 5–7, surgery, postoperative EP, second-line treatment, supportive care	Reported
**10a**	Follow-up and outcomes	Clinician- and patient-assessed outcomes, if available.	pp. 5–8, treatment response and Patient Perspective	Reported; patient-reported benefit was not measured with a validated instrument
**10b**	Follow-up and outcomes	Important follow-up diagnostic and other test results.	pp. 5–7 and 12–13, serial MRI/CT and clinical progression	Reported

Item	Topic	CARE checklist requirement	Reported on page/section	Compliance note
**10c**	Follow-up and outcomes	Intervention adherence and tolerability, including how assessed.	pp. 5–7, adverse effects, dose continuity, and treatment changes	Reported from retrospective clinical records; no dose reduction or toxicity-related treatment delay was documented
**10d**	Follow-up and outcomes	Adverse and unanticipated events.	pp. 5–7, chemotherapy symptoms, anastomotic leak, infection, and urinary complications	Reported: chemotherapy symptoms, no intraoperative complications, postoperative anastomotic leak, infection, and urinary complications
**11a**	Discussion	Strengths and limitations of the approach to the case.	pp. 7–9, Discussion	Reported throughout; limitations include single-case design, absent serial target measurements, unbiopsied lung lesions, and no validated patient-reported instrument
**11b**	Discussion	Discussion of relevant medical literature.	pp. 7–9, Discussion	Reported
**11c**	Discussion	Rationale for conclusions.	pp. 7–9, Discussion	Reported
**11d**	Discussion	Primary take-away lessons in a one-paragraph conclusion without references.	p. 8, Conclusion	Reported
**12**	Patient perspective	The patient shares a perspective on the treatment received.	p. 7, Patient Perspective	Reported retrospectively; no validated questionnaire
**13**	Informed consent	The patient gives informed consent; provide documentation if requested.	p. 3, written informed consent statement	Reported

The manuscript includes a written informed-consent statement for publication. The authors should retain the underlying consent documentation and provide it if requested by the journal.

Checklist completed by: Yang Hang, corresponding author

Date: 22 August 2026
